# Supplementary material for: Arbuscular Mycorrhizal Symbiosis Differentially Affects the Nutritional Status of Two Durum Wheat Genotypes under Drought Conditions
Source: Plants (Basel). 2022 Mar 17;11(6):804. doi: 10.3390/plants11060804 (PMC8954065; doi:10.3390/plants11060804)
Supplement: Supplementary file 1 [file plants-11-00804-s001.zip › plants-1586845-supplementary.pdf]

Table S1. Two-way ANOVA analysis of the morpho-physiological traits, comparisons within genotypes. The effect of a single variable (water status or genotype or AMF) and the variable interaction (water status x AMF) was considered. Significant effects (*p* value < 0.05, 0.01, 0.001) are highlighted in bold.

p-values	Etrusco			Svevo		
	Water status	AMF	Interaction	Water status	AMF	Interaction
RL	*p*>0,05	*p*>0,05	*p*>0,05	*p*>0,05	***p*<0,001**	*p*>0,05
SL	***p*<0,001**	*p*>0,05	*p*>0,05	***p*<0,001**	***p*<0,01**	*p*>0,05
RFW	***p*<0,001**	***p*<0,01**	*p*>0,05	***p*<0,001**	***p*<0,01**	*p*>0,05
SFW	***p*<0,001**	*p*>0,05	*p*>0,05	***p*<0,001**	***p*<0,001**	***p*<0,01**
RWC	***p*<0,001**	*p*>0,05	*p*>0,05	***p*<0,001**	***p*<0,05**	***p*<0,001**

Table S2. Two-way ANOVA analysis of the relative expression of genes involved in water stress response and sulfur assimilation, comparisons within genotypes. The effect of a single variable (water status or genotype or AMF) and the variable interaction (water status x AMF) was considered. Significant effects (*p* value < 0.05, 0.01, 0.001) are highlighted in bold.

p-values	Etrusco			Svevo		
	Water status	AMF	Interaction	Water status	AMF	Interaction
TdSHN1	***p*<0,001**	***p*<0,05**	*p*>0,05	***p*<0,001**	*p*>0,05	***p*<0,05**
TdDRF1	***p*<0,001**	*p*>0,05	***p*<0,05**	***p*<0,01**	*p*>0,05	***p*<0,01**
TdOASTL1	*p*>0,05	***p*<0,01**	*p*>0,05	*p*>0,05	*p*>0,05	*p*>0,05
TdSAT1	***p*<0,05**	***p*<0,05**	***p*<0,01**	***p*<0,05**	***p*<0,01**	***p*<0,01**
TdSULTR1.1	***p*<0,01**	*p*>0,05	***p*<0,01**	*p*>0,05	*p*>0,05	*p*>0,05
TdSULTR1.3	***p*<0,001**	***p*<0,001**	***p*<0,001**	***p*<0,001**	***p*<0,001**	*p*>0,05

Table S3. Two-way ANOVA analysis of the relative expression of genes involved in mycorrhizal colonization The effect of a single variable (water status or genotype) and the variable interaction (water status x genotype) was considered. Significant effects (*p* value < 0.05, 0.01, 0.001) are highlighted in bold.

p-values	AMF		
	Water status	Genotype	Interaction
Fm18S	***p*<0,001**	***p*<0,001**	***p*<0,01**
TdPT11	***p*<0,01**	***p*<0,05**	***p*<0,05**

Table S4. Two-way ANOVA analysis of colonization level parameters. The effect of a single variable (water status or genotype) and the variable interaction (water status x genotype) was considered. Significant effects (*p* value < 0.05, 0.01, 0.001) are highlighted in bold.

p-values	AMF		
	Water status	Genotype	Interaction
F (%)	***p*<0,001**	***p*<0,01**	***p*<0,001**
A (%)	***p*<0,001**	*p*>0,05	*p*>0,05

Table S5. Two-way ANOVA analysis of the cations concentration, comparisons within genotypes. The effect of a single variable (water status or genotype or AMF) and the variable interaction (water status x AMF) was considered. Significant effects (*p* value < 0.05, 0.01, 0.001) are highlighted in bold.

p-values	Etrusco			Svevo		
	Water status	AMF	Interaction	Water status	AMF	Interaction
K^+	*p*>0,05	*p*>0,05	*p*>0,05	***p*<0,001**	***p*<0,001**	*p*>0,05
Na^+	***p*<0,01**	*p*>0,05	*p*>0,05	***p*<0,01**	***p*<0,001**	***p*<0,001**
Ca^{2+}	***p*<0,01**	*p*>0,05	*p*>0,05	***p*<0,001**	*p*>0,05	***p*<0,05**
Mg^{2+}	*p*>0,05	*p*>0,05	*p*>0,05	***p*<0,01**	*p*>0,05	***p*<0,05**
Na^+/K^+	***p*<0,01**	*p*>0,05	*p*>0,05	***p*<0,001**	***p*<0,001**	***p*<0,001**

Table S6. Two-way ANOVA analysis of the anions concentration, comparisons within genotypes. The effect of a single variable (water status or genotype or AMF) and the variable interaction (water status x AMF) was considered. Significant effects (*p* value < 0.05, 0.001, 0.001) are highlighted in bold.

p-values	Etrusco			Svevo		
	Water status	AMF	Interaction	Water status	AMF	Interaction
Cl^-	*p*>0,05	*p*>0,05	***p*<0,001**	*p*>0,05	***p*<0,01**	*p*>0,05
SO_4^{2-}	***p*<0,01**	***p*<0,001**	***p*<0,01**	***p*<0,001**	***p*<0,001**	***p*<0,01**
NO_3^-	*p*>0,05	*p*>0,05	*p*>0,05	***p*<0,001**	***p*<0,001**	***p*<0,001**
PO_4^{3-}	*p*>0,05	***p*<0,05**	*p*>0,05	***p*<0,05**	***p*<0,01**	*p*>0,05
S index	*p*>0,05	***p*<0,01**	***p*<0,001**	*p*>0,05	***p*<0,001**	*p*>0,05

Table S7. Primers used for qRT-PCR analysis.

Gene	Primer name	Sequence
TdSHN1	TdSHN1F	GCTACTCATGAAACTCCACAGTGC
	TdSHN1R	TGGATCTCGACTGGTCAAACCAA
TdDRF1	TdDRF1F	GGGTTCGAAATCGGTTCGGG
	TdDRF1R	CAACACAGGCACCAAGACCG
TdPT11	TdPT11F	TGCATGACACCACGCATCTTATC
	TdPT11R	GGTATCACACACAAGCAATCCTA
Fm18S	Fm18SF	CCTTTTGAGCTCGGTCTCGTG
	Fm18SR	TGGTCCGTGTTTCAAGACG
TdSULTR1.1	TdSULTR1.1F	CTATGAGACAGCCTACCTTATATG
	TdSULTR1.1R	CAACCGCAATGAGCAAGC
TdSULTR1.3	TdSULTR1.3F	ATGTCAGTGGTGGTTCTTC
	TdSULTR1.3R	ATTGCTGCTTCGTAGTCC
TdOASTL1	TdOASTL1F	GCCCAGAGACCCGAGAAC
	TdOASTL1R	AACAGCACCGACGACAGG
TdSAT1	TdSAT1F	GTCTCCATCCTCCACCAC
	TdSAT1R	GCACATCTATCAGCACCAC
GADPH	GADPHF	GGTTTGGCATTGTTGAGGGTTT
	GADPHR	GGAATGATGTTGAAGCTGGCAG

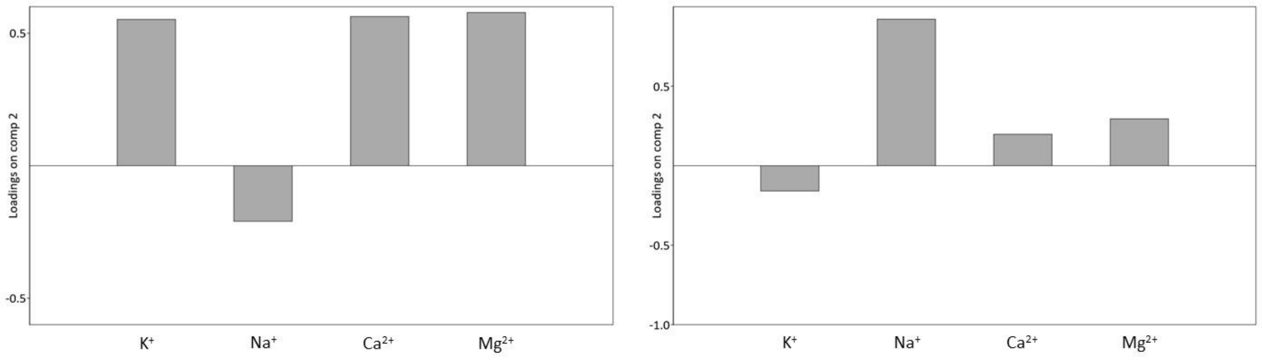

Figure S1. The loading plots of each cations concentration in components 1 and 2, respectively, the bar length representing regression coefficients with either positive or negative signs.

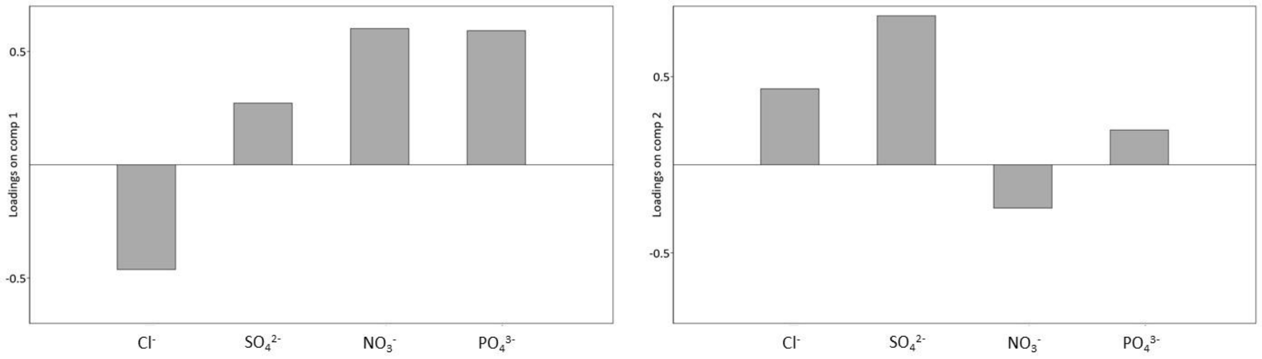

Figure S2. The loading plots of each anions concentration in components 1 and 2, respectively, the bar length representing regression coefficients with either positive or negative signs.
